# The Influence of Fisher Knowledge on the Susceptibility of Reef Fish Aggregations to Fishing

**DOI:** 10.1371/journal.pone.0091296

**Published:** 2014-03-19

**Authors:** Jan Robinson, Joshua E. Cinner, Nicholas A. J. Graham

**Affiliations:** ARC Center of Excellence for Coral Reef Studies, James Cook University, Townsville, Queensland, Australia; The Australian National University, Australia

## Abstract

Reef fishes that exhibit predictable aggregating behaviour are often considered vulnerable to overexploitation. However, fisher knowledge of this behaviour is often heterogeneous and, coupled with socioeconomic factors that constrain demand for or access to aggregated fish, will influence susceptibility to fishing. At two case study locations in Papua New Guinea, Ahus and Karkar islands, we conducted interview-based surveys to examine how local context influenced heterogeneity in knowledge of fish aggregations. We then explored the role of fisher knowledge in conferring susceptibility to fishing relative to socioeconomic drivers of fishing effort. Local heterogeneity in knowledge of aggregating behaviour differed between our case studies. At Ahus, variable access rights among fishers and genders to the main habitats were sources of heterogeneity in knowledge. By contrast, knowledge was more homogenous at Karkar and the sole source of variation was gear type. Differences between locations in the susceptibility of aggregations to fishing depended primarily on socioeconomic drivers of fishing effort rather than catchability. While Ahus fishers were knowledgeable of fish aggregations and used more selective gears, Karkar fishers were less constrained by tenure in their access to aggregation habitat. However, fishing effort was greater at Ahus and likely related to high dependency on fishing, greater access to provincial capital markets than Karkar and a weakening of customary management. Moreover, highly efficient fishing techniques have emerged at Ahus to exploit the non-reproductive aggregating behaviour of target species. Understanding how knowledge is structured within fishing communities and its relation to socioeconomic drivers of fishing effort is important if customary practices for conservation, such as *tambu* areas, are to be supported. The findings of this study call for a holistic approach to assessing the risks posed to reef fish aggregations by fishing, grounded in the principals of fisheries science and emerging social-ecological thinking.

## Introduction

The depletion of reef fish biomass is often attributed to overfishing driven by socioeconomic drivers such as local human population density and distance from reefs to markets [1]–[2]. As changes to these key socioeconomic drivers increase demand for resources, reef fishes with slow life histories, such as groupers (Serranidae), are typically the first to be depleted [3]. However, the rate of depletion will also be influenced by the ability of fishers to locate and exploit fish populations when they are most vulnerable to fishing. Vulnerability to fishing increases when fish aggregate or school and the history of fishing is marked by developments based on exploiting this aspect of fish behaviour [4]–[5]. In the context of coral reefs, the development of aggregation-based fisheries depends on many factors including local knowledge relating to fish behaviour [6]–[7], the technologies available to fishers [8]–[9] and access to aggregation sites [10]–[11]. It is important to understand the key ecological and socioeconomic drivers controlling the evolution of fisheries for aggregating species if they are to be effectively managed.

The exploitation of reef fish spawning aggregations is an obvious example of fishers utilising knowledge on fish behaviour to target populations when their density has increased. A large number of important food fishes on coral reefs aggregate periodically at high density to spawn [12]–[13]. Spawning aggregations represent attractive fishing opportunities since increases in density typically lead to greater catch-per-unit-effort (CPUE) [14] and because they are highly predictable in time and space, as evidenced by acoustic telemetry techniques that reveal spawner fidelity to specific sites and lunar periods [15]–[16]. Predictable aggregating behaviour is not, however, confined to reproduction since reef fishes also aggregate at specific times and locations for other functions, such as foraging, resting and shelter [17]–[18]. Fishers regularly target non-reproductive aggregations [4], [19], though their vulnerability to fishing has received much less research attention than spawning aggregations.

Regardless of their biological predictability, fisher knowledge of aggregations is heterogeneous and will influence the extent to which aggregations are perceived as predictable and exploited by fishers. Fisher knowledge maybe stratified by factors such as gender, age, location and cultural background [6], [13], [20]. For example, Hamilton et al. (2004) [6] documented how fisher knowledge of spawning aggregations varied by clan both within and between locations in Manus Province, Papua New Guinea. Even if aggregations are predictable and their timing and location are known to fishers, accessibility to sites may be low due to factors such as prevailing weather and remoteness [10]–[11], while inefficient gear use may constrain exploitation rates [21]. Gender preferences and customary marine tenure that specifies ownership rights among kinship groups may also influence fisher access to fish resources [7], [22]. Furthermore, fishing effort on aggregations may be constrained by limited market access or fish preservation capacity [10]. Consequently, the vulnerability to fishing conferred by aggregation formation will depend on both fisher knowledge of aggregating behaviour and socioeconomic drivers influencing aggregation exploitation.

Fisher knowledge of fish aggregating behaviour will be influenced by cognitive processes (such as recall) and the formation of heuristic models [23]. To understand how such knowledge develops, it is informative to deconstruct the biological attributes of this behaviour and consider their effects on fisher memory. The biological attributes of aggregation behaviour can be categorised by their temporal, spatial and physical manifestations. Firstly, aggregation formation aligning with diurnal, lunar and seasonal periods is likely to promote recall since coral reef fishers often allocate effort according to such schedules [24]. Secondly, reef fishers often have detailed knowledge on the broad-scale (i.e. seascape) distribution of resources [7], which coincides with the fact that aggregations often form at prominent reef features [25]–[26]. However, some species are more mobile and therefore less predictable in space than others when aggregated for spawning (e.g. Carangidae) [27]. Lastly, the size of aggregations formed is expected to influence recall since memory varies according to how pleasurable, unusual or emotive an experience is [28], while the presence of eggs or milt (i.e. spawning) are physical manifestations of behaviour that enable fishers to reconcile aggregation formation with biological function. In combination, these attributes are expected to influence the extent to which fishers develop knowledge on aggregations and perceive them as predictable.

Assessing the status of aggregating reef fish populations is problematic due to the data-poor context of their fisheries [29]. Vulnerability assessment frameworks developed for data-poor contexts, which combine measures of a species productivity and susceptibility to a fishery, (e.g. [30]–[31]), are therefore worth examining for such species. Productivity defines the capacity of a stock to recover rapidly following depletion, while susceptibility is the potential for the stock to be impacted by the fishery [31]. Measures of productivity are generally available for reef fishes through empirical tools based on life history invariants [32], whereas indicators of susceptibility can be tailored to the specific fisheries being assessed in terms of patterns in catchability and socioeconomic drivers [31]. Thus, a population's susceptibility to aggregation fishing will be governed by catchability that, among other factors, relates to the accessibility of the aggregation site and the selectivity or efficiency of gears used at the site [33]. Catchability will in turn be driven by socioeconomic drivers, such as market access and dependency on fishing, that influence technological development and fishing effort [2], [34]. However, aggregations are often transient phenomena, particularly in the case of spawning aggregations [12], and fisher knowledge of their dynamics should be considered a critical component of susceptibility to fishing. Fisher knowledge will effectively act as the basis for the development of an aggregation-based fishery, the trajectory of which is subsequently affected by catchability attributes and socioeconomic drivers.

Studies documenting fisher knowledge of reef fish aggregations have primarily gathered information in order to identify research, conservation and management priorities [35]. Attempts to quantify the influence of fisher knowledge in the susceptibility of populations to aggregation fishing are lacking, as is the use of indicator-based vulnerability frameworks for these fisheries. In this study, we aimed to examine how fisher knowledge of reproductive and non-reproductive aggregations influences the susceptibility of populations to fishing at two case study sites in Papua New Guinea. The specific research questions were: (1) to what extent are fishers knowledgeable of aggregations and do they perceive them as predictable?; (2) how does variation in fisher knowledge of aggregations relate to local socioeconomic indicators?, and (3), what is the influence of fisher knowledge in conferring susceptibility to fishing relative to catchability and socioeconomic drivers of fishing pressure.

## Materials and Methods

### Ethics statement

The study was approved by the Human Ethics Research Committee of James Cook University (Ethics Approval Number H4812). A permit was obtained for research in Papua New Guinea (Permit number 10350012505). Due to low levels of literacy at our study locations, the Human Ethics Research Committee approved for fishers to provide verbal consent to participate in the study, according to the following procedures. An information sheet detailing the aims of the research and how data were to be used was translated verbally to participants, which also specified their rights to withdraw their information at any time and guaranteed their anonymity as participants. This was followed by verbally translating a consent form to participants. Upon consent, the consent form was signed by the lead author (JR) and translator, copies of which are stored with the unprocessed data at James Cook University. For follow-on contact with the lead author, copies of the information sheet were left with clan leaders at each location.

### Study locations and communities

**Figure 1:**
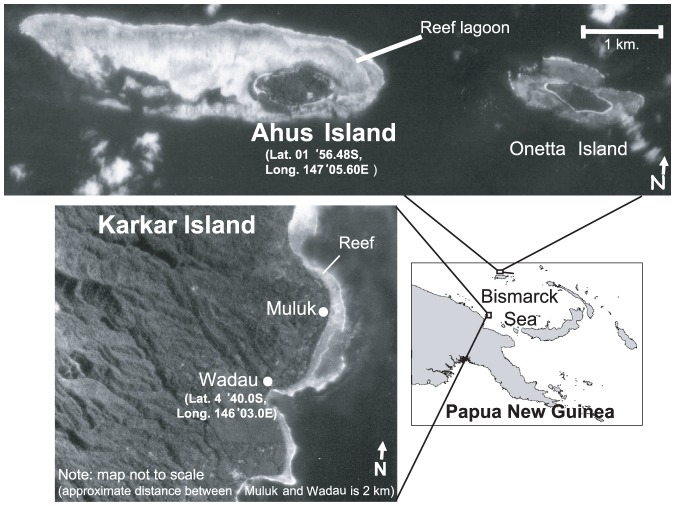
Study locations. Papua New Guinea with details (insets) of Ahus Island, Manus Province, and Karkar Island, Madang Province.

We studied reef fisheries at two locations in Papua New Guinea, representing two extremes of fishing pressure and comprising gear and fishing practices common to the region [22]. Studies focused on the communities of Ahus Island (Manus Province) and Muluk and Wadau villages, Karkar Island (Madang Province) (Figure 1). Karkar is a large, elevated (1,839 m) volcanic island and fishing is a secondary occupation to agriculture. By contrast, fishing is the primary occupation for the community on the small (28 ha), low-lying Ahus Island where terrestrial resources are limited [22]. The two locations also differ in coastal geomorphology and habitats. Fishers from Karkar have access to a narrow (<1 km) fringing reef system of less than 150 ha with a narrow lagoon [22], [36], whereas fishers at Ahus Island are surrounded by a wide (>4 km in the west), extensive lagoon system of approximately 550 ha [22].

Fishers at the study sites use a combination of gear types including line, net and spears. Fishing effort at Ahus primarily comprises use of lines and spearguns (97%, of total fishing effort), whereas effort at Karkar comprises both of these gears (72%) in combination with hand spearing (24%). By comparison, a small proportion (<4%) of fishing effort at both study sites involves use of nets [22]. Resource use is governed by a system of customary marine tenure (CMT) that recognises local ownership of inshore marine resources. Tenure in Karkar is a relatively centralised approach where governance is controlled by a council of chiefs. There is relatively high mobility, with fishers having the ability to switch between gears and fishing grounds [22]. By contrast, tenure at Ahus is highly decentralized and access to fishing grounds and gears (particularly nets) is controlled by kinship group (individuals, families, clans). Both study communities have traditionally used customary taboos (*tambu*) to restrict fishing in certain areas in an effort to influence catchability (i.e. make fish less wary of spear fishers) or rebuild biomass for feasts [36]–[37].

### Quantifying fisher knowledge of aggregating behaviour and predictability

Interviews were conducted with fishers at Ahus (n = 16) and Karkar (Muluk: n = 7; Wadau: n = 9) in October 2012 to quantify fisher knowledge on aggregating behaviour and to develop an index of knowledge pertaining to fisher perceptions of aggregation predictability. At Karkar, interviews were conducted with all fishers for whom fishing was a regular livelihood activity. In Ahus, where the proportion of residents engaged in fishing was high by comparison, a sample of fishers, representative of fisher gender, clan membership and gear use, was taken. After pilot studies (n = 4) with Karkar fishers, a semi-structured questionnaire was designed to investigate fisher knowledge of the form, function and predictability of aggregating behaviour for six species of reef fish common to fisheries in both locations. The six species comprised two groupers (*Epinephelus fuscoguttatus*, *Plectropomus areolatus*), two emperors (Lethrinidae; *Lethrinus harak*, *L. lentjan*) and two snappers (Lutjanidae; *Lutjanus fulviflamma*, *L. gibbus*). All species are high tropic level predators (trophic levels 3.6–4.5) and were selected to contrast forms and functions of aggregating behaviour (Table 1).

**Table 1:** Evidence on aggregating behaviour of study species.

Species	Aggregating behaviour
*Epinephelus fuscoguttatus*	Primarily solitary and territorial; form large aggregations for spawning^a^^, ^ b ^, ^ c ^, ^ d
*Plectropomus areolatus*	Primarily solitary and territorial; form large aggregations for spawning^a^^, ^ b ^, ^ c ^, ^ e
*Lutjanus gibbus*	Primarily schooling; forms large aggregations for spawning^a^^, ^ b ^, ^ c
*Lutjanus fulviflamma*	Primarily schooling; spawning aggregation formation not verified^a^^, ^ c ^, ^ f
*Lethrinus lentjan*	Primarily solitary as adults; spawning aggregation formation suspected but not verified^a^^, ^ b ^, ^ c ^, ^ g
*Lethrinus harak*	Primarily solitary or forms small groups (<10 fish); spawning aggregation formation suspected but not verified^g^^, ^ h

a Sadovy de Mitcheson et al. (2008);

b Claydon (2004);

c Froese and Pauly (2003);

d Robinson et al. (2008);

e Rhodes and Tupper (2008);

f Grandcourt et al. (2006).

g Ebisawa (2006);

h Nanami and Yamada (2009).

Firstly, species recognition by fishers was established using a combination of pictures (landed specimens) and local names specific to location. Secondly, we asked fishers whether they caught each species frequently, infrequently or not at all and, if they encountered the species, to estimate the ‘poor’, ‘normal’ and ‘good’ catch rates (fish.trip^−1^) that they typically obtain for each species when using their primary gear. Thirdly, fishers were questioned on their knowledge of aggregating behaviour, initially focusing on whether they observe a species to display solitary, shoaling (groups of three or more fish displaying unsynchronised swimming) and schooling (groups of three or more fish displaying synchronised swimming) behaviour [5]. Fishers could assign multiple behavioural types to each species, giving eight potential categories including a ‘don't know’ response. For example, a species could be identified as solitary and shoaling, or as displaying all three behaviour types. Unless specified, ‘aggregation’ refers to all forms (i.e. shoaling, schooling) and functions (e.g. resting) of social group behaviour.

Seven attributes of the spatial, temporal and physical manifestations of aggregating behaviour were employed to quantify fisher knowledge of aggregations and their perceived predictability (Table 2). Aggregation attributes were discussed for each species and scored according to fisher responses. For some statistical tests and analyses (*see below*), an index of fisher knowledge of aggregating behaviour was calculated for each fisher and species by summing the scores for the seven aggregation attributes (maximum score = 17; Table 2). Thus, the fisher knowledge index essentially aims to measure the predictability of aggregations as perceived by fishers. For example, to obtain a maximum score, a fisher would need to recognise that aggregations form consistently at specific locations within a small home range, that formation aligns with diel, lunar and seasonal schedules, that aggregations are large (>500 fish), and that they form for spawning (Table 2). Aggregation size and spawning (presence of eggs or milt) are physical manifestations of behaviour that were assumed to promote recall and therefore perceived predictability. Since some species were reported as constantly shoaling or schooling, questions on periodicities of aggregation formation were obviously irrelevant. Therefore, fishers were asked as to whether aggregation size in frequently shoaling or schooling species (e.g. *L. gibbus*) increased with spawning.

**Table 2:** Scoring of aggregation attributes based on fisher knowledge of aggregating behaviour.

Attribute	Scoring^a^
Aggregation size	1 = solitary or pairing
	2 = aggregations of 3–10 fish
	3 = aggregations of 10–100 fish
	4 = aggregations of 100–500 fish
	5 = aggregations larger than 500 fish
Aggregation location	1 = aggregation location is unknown or variable
	2 = aggregations form in specific areas of the reef
Home range	1 = species of high mobility and occupying large home range
	2 = species of low mobility and occupying small home range
Spawning	1 = aggregation formation not associated with spawning (eggs/milt absent)
	2 = aggregation formation associated with spawning (eggs/milt present)
Diel	1 = aggregation formation not aligned with time of day
	2 = aggregation formation aligned with particular time of day
Lunar	1 = aggregation formation not aligned with lunar phase
	2 = aggregation formation aligned with particular lunar phase
Seasonal	1 = aggregation formation not aligned with month or season
	2 = aggregation formation aligned with particular month or season

a If fishers had no knowledge of an attribute, a zero score was given.

### Socioeconomic indicators related to heterogeneity in fisher knowledge within study locations

To investigate sources of variation in fisher knowledge within case study locations, data relating to socioeconomic indicators were collected during interviews. Indicators were selected based on literature pertaining to sources of variation in knowledge among coral reef fishers and included gender, dependency on fishing as a livelihood, and access rights to major reef habitats and gear types (Table 3). Gender and the main reef habitats for which fishers hold access rights often structure fisher knowledge of fish behaviour [6]–[7], [20]. Fishing gears vary in species selectivity and the habitats where they can be deployed, influencing the potential for fishers to capture and develop knowledge of species behaviour [38]. Finally, dependency on fishing influences levels of fisher knowledge [39] and was derived from fisher rankings of the importance of fishing as a livelihood (primary, secondary, tertiary) (Table 3).

**Table 3:** Socioeconomic indicators used in redundancy analysis (RDA) to identify sources of variation in fisher knowledge of aggregating behaviour.

Factor	Measurement level or category	Ahus	Karkar
Fisher gender	Male	12	15
	Female	4	1
Fisher access to habitat	Lagoon only	5	0
	Outer reefs only	4	0
	All habitats	7	16
Primary gear type	Line	9	13
	Speargun	6	2
	Net	1	1
Dependency on fishing	Primary	13	2
	Secondary	2	7
	Tertiary	1	7

Data are the number of fishers scored at each factor level or category for case study locations.

### Fisher knowledge and the susceptibility of aggregations to fishing

A productivity-susceptibility analysis (PSA) using the bivariate framework of Hobday et al. (2011) [30] was employed to determine the relative importance of fisher knowledge in conferring susceptibility of aggregations to exploitation, and to assess the overall risk to populations posed by aggregation fishing. PSA reduces life history parameters associated with species productivity to a single x-axis index and susceptibility attributes to an index on the y-axis. In line with the approach of Hobday et al. (2011) [30], we scored seven life history parameters (Table 4) for the six species, where productivity score categories are: 1 = high, 2 = moderate and 3 = low productivity. Parameter estimates for each species were derived using the life-history tool of Fishbase.org [32]. Cut-off points dictating membership of each productivity category were adopted from those used for fisheries of the United States, which include fisheries for reef fishes analogous to those of Papua New Guinea [31]. Fecundity was subsequently omitted from the index since data were lacking for the study species.

**Table 4:** Attributes and their scoring system employed for productivity-susceptibility analysis (PSA).

Category	Attribute	Scoring
Productivity	Average age at maturity	1: <2 years; 2: 2–4 years; 3: >4 years
	Average maximum age	1: <10 years; 2: 10–30 years; 3: >30 years
	Average size at maturity	1: <30 cm; 2: 30–50 cm; 3: >50 cm
	Average maximum size	1: <60 cm; 2: 60–150 cm; 3: >150 cm
	Reproductive strategy	1: broadcast spawner; 2: demersal egg layer; 3: live bearer
	Trophic level	1: <2.5; 2: 2.5–3.5; 3: >3.5
Catchability	Fisher knowledge index	1: index scores 1–6; 2 :index scores 7–12; 3: index scores 13–17
	Availability	Proportion of fisher's effort allocated in aggregation habitat, 1: none; 2: some; 3: all
	Encounterability	Species occurrence in fisher's catch, 1: never; 2:infrequently; 3: frequently
	Selectivity	1: gears unselective for species; 2: uses one of the two most selective gears; 3: uses both of the two most selective gears
Socio-economics	Habitat impact of gear	1: fisher uses spear guns and/or hook-and-line; 2: fisher uses gill nets; 3: fisher uses scare lines
	Fishing effort	1: 10–50 hrs/month; 2: 75–120 hrs/month; 3: 145–265 hrs/month
	Preference	1: low and medium preference spp.; 2: high preference spp.; 3: very high preference spp.
	Dependency on fishing	Importance of fishing as an occupation, 1: tertiary; 2: secondary; 3: primary
	Management strategy	Ownership rights and conservation measures (i.e. closures), 1: both exist; 2: one or the other exists; 3: none exist at study location
	Access to markets*	1: Sell/barter catch in village; 2: sell/barter catch in neighbouring villages; 3: sell/barter catch in provincial capital markets.

Susceptibility attributes are subdivided into attributes associated with catchability and those associated with socioeconomic drivers of fishing pressure or habitat impacts.

*: The provincial capital markets for Karkar and Ahus are Madang (Madang Province) and Lorengau (Manus Province), respectively.

The four susceptibility attributes of Hobday et al. (2011) [30] were adapted to address the susceptibility of populations to aggregation fishing. Firstly, the attribute of availability primarily concerns the overlap (spatial and depth) between fishing effort and species or population distribution, or in our case the access fishers have to habitats where aggregations or schools are perceived to occur (Table 4). Secondly, the attribute of encounterability concerns the likelihood that a specific gear will encounter aggregated fish if sites are available to fishers. Fisher responses to the question on whether they catch the species frequently, infrequently or not at all were used as a measure of encounterability, assuming that the gears used by that fisher would be as efficient in catching the fish while aggregated. Thirdly, to measure selectivity, i.e. the potential of the gear to capture and retain species, we used fisher reports of catch rates (fish.trip^−1^). The mean ‘good’ catch rate across fisher responses was taken on each gear used for a species, from which gear selectivity was ranked by order of catch rate. Fourthly, we replaced post-capture mortality [30], which is less relevant to small-scale reef fisheries where discards are minimal [40], with our fisher knowledge index (described above). All susceptibility attributes were scored from 1 to 3, with 1 indicative of low susceptibility and 3 of high susceptibility (Table 4).

The attributes of Hobday et al. (2011) [30] relate to potential for a fishery to access, encounter and select for a species, i.e. catchability. However, we also wanted to quantify fishing effort (e.g. days fished each month), which in combination with catchability will determine the fishing pressure (i.e. mortality rate) on resources, and explore the socioeconomic drivers of that fishing effort. Informed by known drivers of fishing pressure in reef fisheries [2], [34], [41] and several of the ‘management attributes’ employed by Patrick et al. (2010) [31], which also equate to socioeconomic drivers, we developed six additional susceptibility attributes. These were habitat impact of gear, fishing effort, preference for the species, dependency on fishing for a livelihood, management strategy and access to markets (Table 4). While catchability attributes were scored for each species and fisher, the socioeconomic attributes were not species-specific and combined fisher and location-level scoring (Table 4). A ranking of habitat impacts associated with gears used by fishers in our study was developed from Mangi and Roberts (2006) [42] and Corpuz et al. (1985) [43]. Fishing effort was quantified for each fisher by questioning the hours and days they fish each day and week, respectively, which was converted to hours fished per month. Ranges associated with low, medium and high susceptibility was estimated by cluster analysis of individual effort reported by fishers. Preference, a location-level attribute used as a proxy for desirability or value of the species [31], was derived for our six study species from a focus group held in each community. Dependency on fishing and access to markets are significant drivers of fishing pressure in many reef fisheries [2], [41] and were derived from individual fisher rankings of the importance of fishing as a livelihood (*as detailed above*) and markets that they access, respectively. Management strategy was a location-level attribute adopted from Patrick et al. (2010) [31] but modified for the local context (Table 4) [22].

### Data analysis

The mean ‘normal’ catch rate estimated by fishers for each species was compared between locations using a *t*-test, assuming unequal variances. For each species, associations between fisher knowledge on aggregation form at Ahus and Karkar were analysed by constructing contingency tables of the frequency of observation for each of the eight categories (solitary, shoaling, schooling, combinations of the three forms, and the ‘don't know’ response). Cramér's *V* contingency coefficient was used as the measure of association; the coefficient ranges between 0 (no association) and 1 (perfect association). Since expected frequencies were less than five for a high proportion of cells in the contingency table, *p*-values were calculated using Monte Carlo simulation (10,000 sampled tables).

Several methods were employed to examine how fisher knowledge of aggregations varied by location and species. Firstly, for each species and aggregation attribute, fisher respondent scores were averaged in each location and the difference between the averages (Ahus minus Karkar) plotted. Secondly, Mann-Whitney U tests were used to compare fisher responses in each location, again for each species and attribute, with exact significance (2-sided) *p*-values reported rather than asymptotic values due to small sample sizes (n≤17). Owing to the risk of type 1 errors arising from multiple comparisons, *p*-values were adjusted with a false discovery rate (FDR) correction for multiple testing using the Benjamini-Hochberg method [44]. To make comparisons between locations, the fisher knowledge index was averaged across fishers for each location and the same statistical method as that applied to individual aggregation attributes was used. For statistical tests of variation in both individual aggregation attributes and in the fisher knowledge index, comparisons were restricted to those fishers knowledgeable on the species and its behaviour (i.e. excluding fishers that do not catch the species).

Redundancy analysis (RDA), which combines concepts of ordination and regression [45], was used to examine the relationship between socioeconomic indicators (Table 3) and variance in fisher knowledge. RDA was conducted separately for each case study as the two locations differ significantly in their socioeconomic conditions and the aim was to examine local sources of variation in fisher knowledge. Here, all fisher respondents for a location were included in the analysis (n = 16) since variation in knowledge was integral to the analysis. However, results for Ahus should be treated with caution since sample size relative to the number of indicator (factor) levels imposed limitations on the RDA. Access to habitat was not included in the RDA for Karkar since it did not vary among respondents.

Estimates of productivity attributes were averaged to give a single productivity score per species. To assess the relative importance of catchability and socioeconomic drivers of fishing pressure for the six species, each susceptibility attribute was first scored for each fisher respondent individually and then averaged across fishers to give a single attribute score for each location. Within location, the susceptibility attributes were combined by averaging across two sets of attributes; (1) the full set of 10 susceptibility attributes, and (2) the four catchability attributes (Table 4). Consequently, two bivariate PSA plots were produced for the 12 fish populations (six species per location), one for productivity and the full set of susceptibility attributes, and a second for productivity and using only the four catchability attributes (we only present the PSA plot for the full set of susceptibility attributes). From both of these PSA plots, overall vulnerability (or risk) was derived for each population at Ahus and Karkar by taking the Euclidean distance between the origin and population location in the bivariate space [30]. Wilcoxon Signed Rank tests were used to determine if overall vulnerability (with species as paired samples and location as treatments) differed between Ahus and Karkar for the full susceptibility attribute set and for the catchability attributes. Exact significance (2-sided) *p*-values are reported due to the small number of paired-samples (n = 6).

## Results

**Figure 2:**
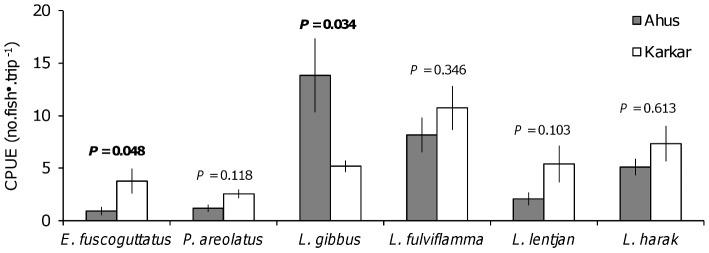
Catch rates reported by fishers for the six study species. Data are the ‘normal’ catch rates fishers expect to obtain on a fishing trip, given as mean no. fish/trip^−1^ with standard error bars. For each species, results of *t*-tests comparing mean catch rates between locations are shown, with significant differences indicated by bold font.

A greater proportion of females engaged in fishing at Ahus and fishers were more dependent on fishing for a livelihood than their counterparts from Karkar (Table 3). Lines were the dominant gear type in both locations but a greater proportion of fishers from Ahus used spearguns as their primary gear. Contrasting with Karkar, some fishers from Ahus reported that they were limited to fishing in the lagoon or on the outer reefs (Table 3). Based on the median response among fishers, groupers were encountered infrequently in the catches at both study locations. The four species of snapper and emperor were encountered frequently in the catches of Karkar fishers, while at Ahus *L. gibbus* and *L. harak* were encountered frequently and *L. fulviflamma* and *L. lentjan* infrequently, again based on median fisher response. Reported catch rates of snappers tended to be greater than those of emperors and groupers (Figure 2). Comparing between locations, the mean reported catch rates did not differ for most species. However, the mean catch rate for *E. fuscoguttatus* was greater at Karkar, while the opposite was true for *L. gibbus*.

**Figure 3:**
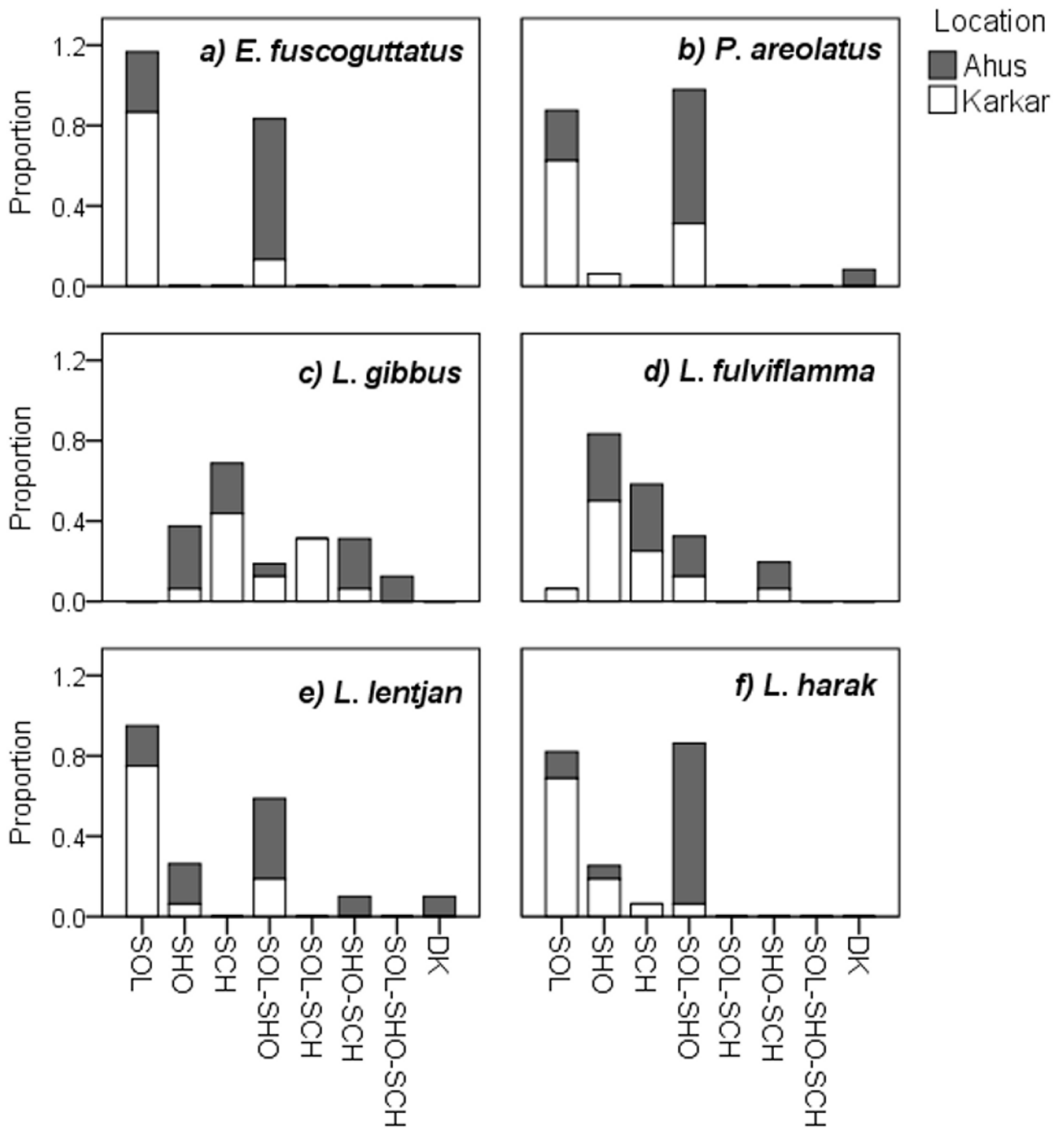
Fisher knowledge of the aggregating behaviour of study species. Stacked bars represent the proportion of fishers in Ahus and Karkar identifying the six study species as exhibiting solitary (SOL), shoaling (SHO) and schooling (SCH) behaviour, or any combinations thereof (SOL-SHO, SOL-SCH, SHO-SCH). DK denotes the proportion of fishers who didn't know the aggregating behaviour of the species.

Fishers from Ahus and Karkar had different perceptions on the forms of aggregating behaviour exhibited by the two groupers (*E. fuscoguttatus*: *V* = 0.237, *p* = 0.584; *P. areolatus*: *V = *0.393, *p* = 0.335). The groupers were primarily perceived as solitary by fishers from Karkar while a larger proportion of Ahus fishers recognised that they are generally solitary but also form spawning aggregations (Figure 3a,b). By contrast, fishers from both Ahus and Karkar perceived snapper aggregating behaviour to be complex, alternating between solitary occurrence, loose shoal formation and synchronised schooling (Figure 3c,d). In spite of this complexity, there were significant associations between locations in how fishers perceived the forms of aggregating behaviour (*L. gibbus*: *V* = 0.708, *p* = 0.009; *L. fulviflamma*: *V = *0.674, *p* = 0.025). Emperor aggregating behaviour was also considered more complex than that of groupers, encompassing reports of schooling by two fishers, but also tended towards solitary and shoaling behaviour (Figure 3e,f). Aggregation formation was considered a much more common behaviour by fishers on Ahus, especially for *L. harak*. As with the two groupers, the null hypothesis of no association between locations in perceived behaviour was accepted for the lethrinids (*L. lentjan*: *V* = 0.732, *p* = 0.07; *L. harak*: *V* = 0.229, *p* = 1.0).

**Figure 4:**
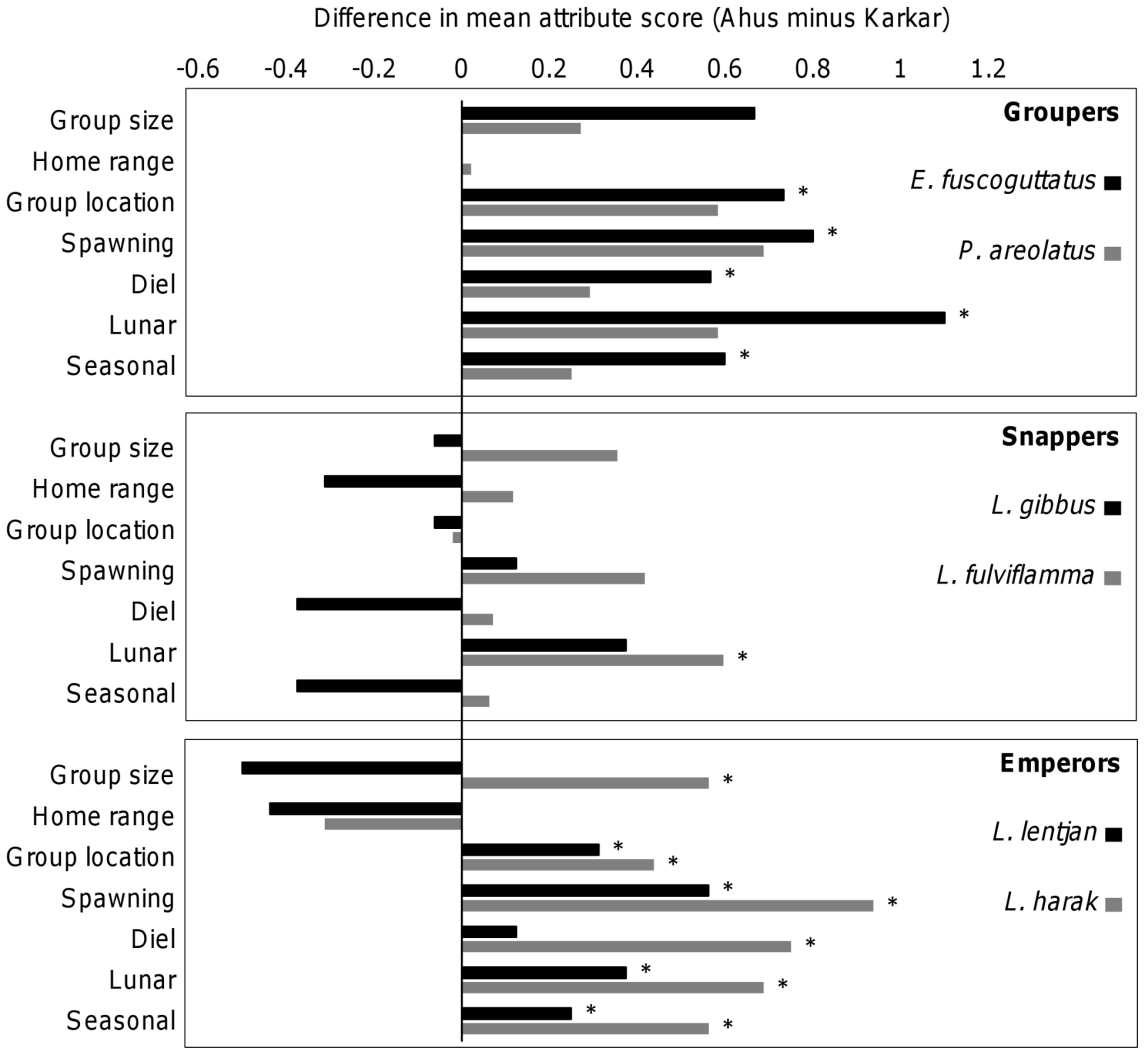
Differences in fisher knowledge of aggregation attributes between study locations. For each species and location, attribute scores were averaged among fishers. Average The results of statistical tests (Wilcoxon Signed Rank tests) comparing the vulnerability of populations (species pooled) at Ahus and Karkar are given in the panel titles.

Ahus fishers were generally more knowledgeable on the seasonal, lunar and diel periodicity of aggregations for *L. lentjan*, *L. harak* and *E. fuscoguttatus* (Figure 4). By contrast, Ahus and Karkar fishers did not differ statistically in their knowledge of aggregation attributes for *L. gibbus* and *P. areolatus*, while significant differences for *L. fulviflamma* were limited to a greater knowledge of aggregation lunar timing among Ahus fishers. Fisher knowledge of aggregation formation or increased catchability being associated with spawning was also more common on Ahus for *L. lentjan*, *L. harak* and *E. fuscoguttatus*. Moreover, aggregation locations for these three species were perceived as more predictable by Ahus fishers. There were no differences between Ahus and Karkar in the perceived home range sizes of the six species and the only species for which perceptions of aggregation size differed significantly was *L. harak*, with larger aggregations perceived on Ahus (Figure 4).

**Figure 5:**
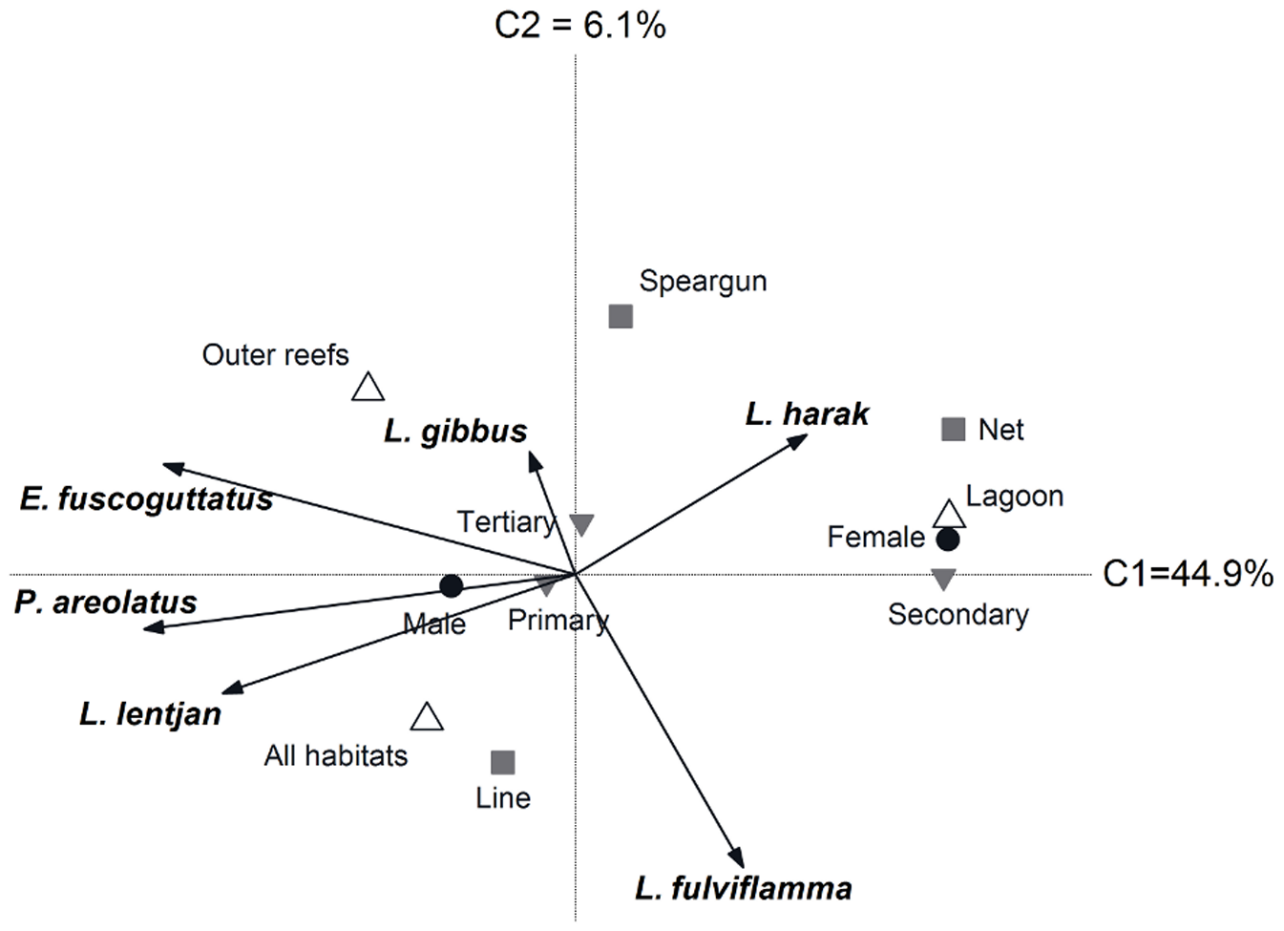
Socioeconomic indicators associated with variation in fisher knowledge at Ahus Island. A redundancy analysis plot of fisher knowledge relating to aggregating behaviour of six study species. Indicators are fisher gender (black circles), fisher access to lagoon, outer reef or all habitats (white triangles), primary gear type (grey squares) and primary, secondary or tertiary dependency on fishing (inverted grey triangles).

For Ahus, ordination of species by fisher knowledge of their aggregating behaviour loosely clustered *L. lentjan*, *E. fuscoguttatus* and *P. areolatus*, separating them from the other species along the first canonical axis that accounted for 44.9% of the variation (Figure 5). Knowledge of the aggregating behaviour of these three species was primarily held by male fishers, whereas female and net fishers who fish in the lagoon were more knowledgeable of *L. harak* (Table 5). Knowledge of snapper (*L. gibbus* and *L. fulviflamma*) aggregating behaviour was largely shared among fishers, with the species orientated on the second canonical axis that only accounted for 6.1% of the variation. Therefore, with the exception of snappers, knowledge of species aggregating behaviour at Ahus was heterogeneous and explained by fisher gender and right of access to the major reef habitats (Table 5).

**Table 5:** Variation in fisher knowledge of aggregating behaviour explained by socioeconomic indicators.

	Ahus		Karkar	
Factor: factor level	Explains (%)	*P*	Explains (%)	*P*
Fisher gender: male	31.4	**0.004**	4.8	0.582
Fisher gender: female	31.4	**0.008**	4.8	0.534
Access to habitat: lagoon only	43.1	**0.002**		
Access to habitat: outer reefs only	11.6	0.128		
Access to habitat: all habitats	13.8	0.084		
Primary gear type: line	8.9	0.266	10.2	0.124
Primary gear type: speargun	4.8	0.542	20.3	**0.026**
Primary gear type: net	6.7	0.472	5.2	0.494
Dependency on fishing: primary	3.3	0.662	4.4	0.644
Dependency on fishing: secondary	6.5	0.532	8.9	0.27
Dependency on fishing: tertiary	1.1	0.89	12.4	0.084

Redundancy analysis (RDA) results for Ahus and Karkar with significant *p*-values highlighted in bold. Access to habitat did not vary among Karkar fishers and was not included in the RDA.

**Figure 6:**
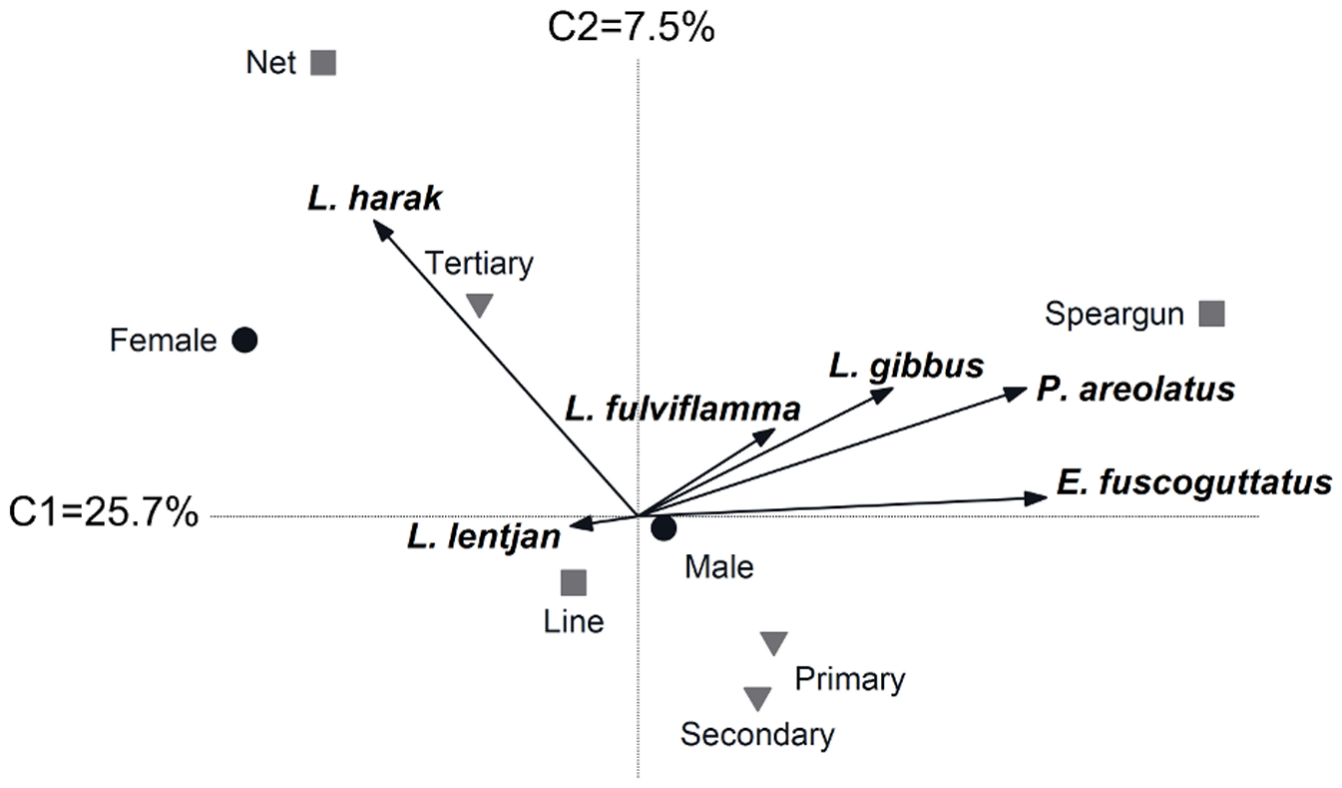
Socioeconomic indicators associated with variation in fisher knowledge at Karkar Island. A redundancy analysis plot of fisher knowledge relating to aggregating behaviour of six study species. Indicators are fisher gender (black circles), primary gear type (grey squares) and primary, secondary or tertiary dependency on fishing (inverted grey triangles).

The RDA explained less of the variation in fisher knowledge at Karkar compared to Ahus, with axes one and two accounting for 25.7% and 7.5%, respectively (Figure 6). Knowledge of grouper (*E. fuscoguttatus* and *P. areolatus*) aggregating behaviour again clustered on the first canonical axis but was restricted to only two fishers primarily using spearguns. Use of this gear constituted the only factor that significantly explained variation in knowledge (Table 5). *Lutjanus fulviflamma* and *L. gibbus* orientated between the axes, again due to knowledge being largely shared among fishers, while the low level of knowledge pertaining to *L. lentjan* behaviour was not influential on the ordination. Line and net fishers with a tertiary level of dependence on fishing were knowledgeable of *L. harak*, but these factors were not statistically significant in explaining variation in knowledge (Table 5).

**Figure 7:**
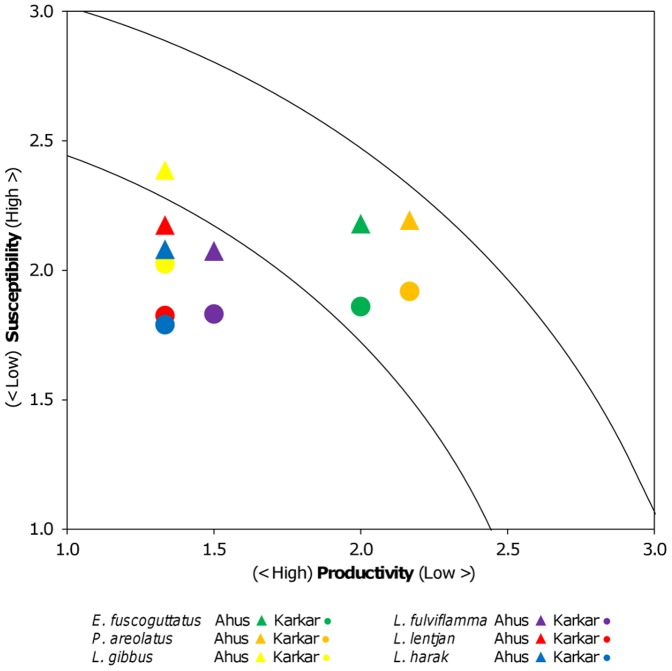
Productivity-susceptibility analysis (PSA) plot. The susceptibility axis in this plot combines all ten attributes associated with catchability and socioeconomic drivers of fishing pressure (see Table 4). The contour lines divide regions of equal vulnerability to fishing, and group species of similar risk levels: i.e. low, medium and high risk (after Hobday et al. 2011).

Productivity of the six species varied from less productive groupers to the more productive emperors and snappers. Three species (*L. harak*, *L. lentjan* and *L. gibbus*) were equal in their productivity scores (Figure 7). All four grouper populations were in the medium risk category, while most of the other populations were low risk. However, *L. gibbus* aggregations at Ahus were assessed to be medium risk due to a high susceptibility score.

**Figure 8:**
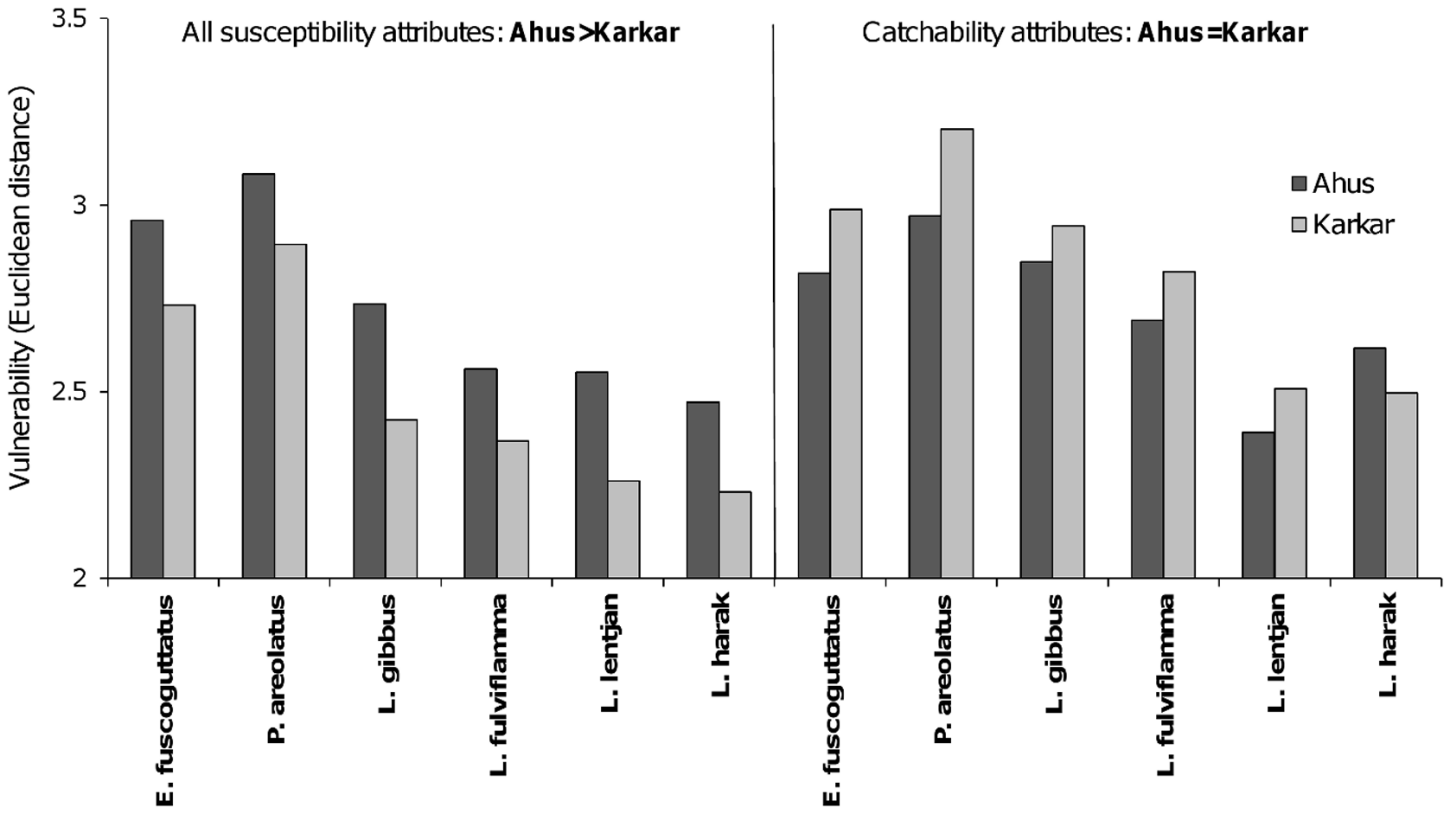
Influence of catchability and socioeconomic susceptibility attributes on the vulnerability of populations to aggregation fishing. Vulnerability, measured as the Euclidean distance of populations to the origin in the corresponding PSA plots, is compared for all susceptibility attributes (catchability and socioeconomic drivers; left panel) and catchability (right panel) attributes only. The results of statistical tests (Wilcoxon Signed Rank tests) comparing the vulnerability of populations (species pooled) at Ahus and Karkar are given in the panel titles.

**Figure 9:**
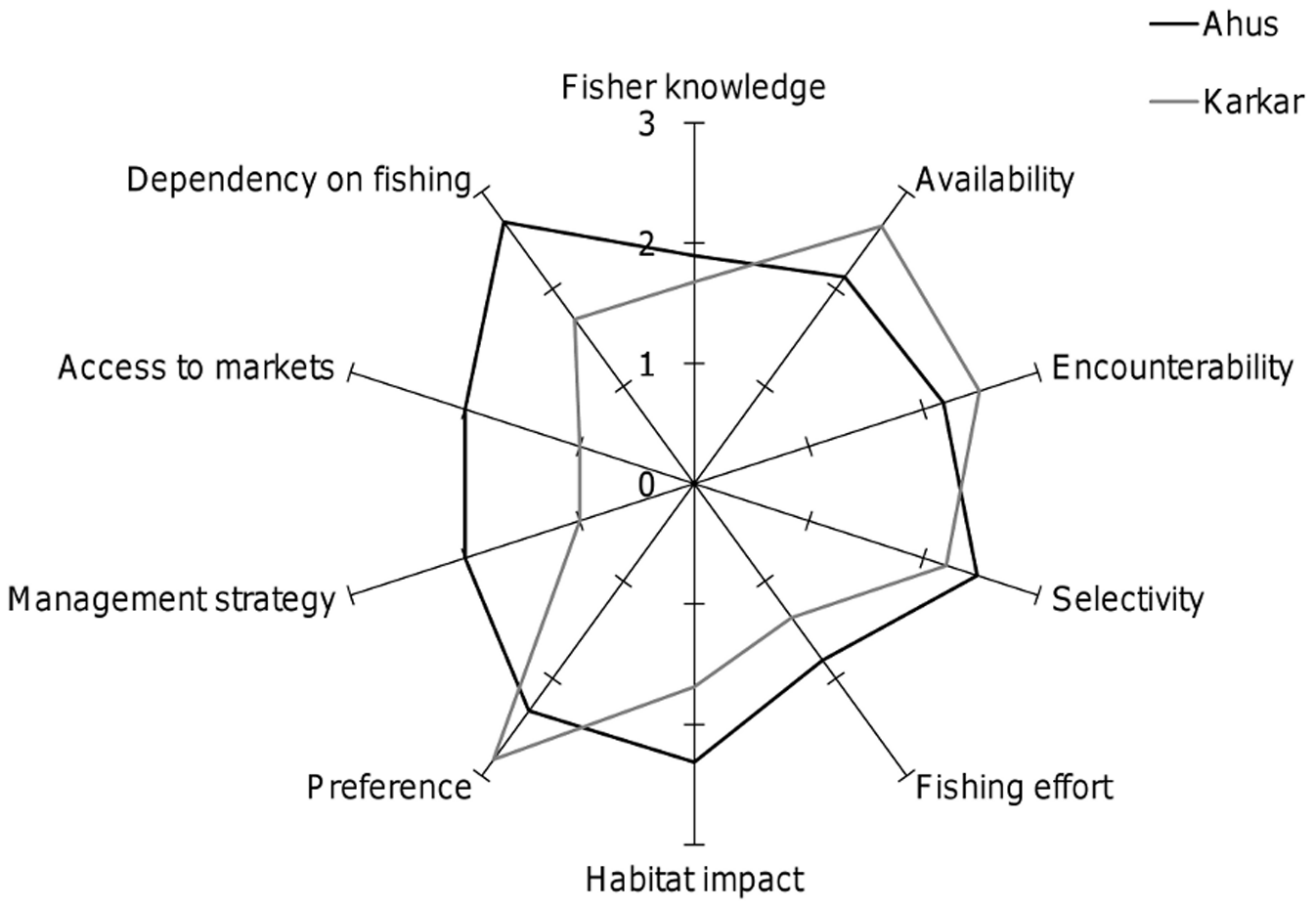
Case study location scores for the ten susceptibility attributes. Clockwise from top, the first four attributes relate to catchability, while the remaining six are indicators of socioeconomic drivers. Attributes were scored from 1 (low susceptibility) to 3 (high susceptibility) for each fisher respondent or, in the case of preference and management strategy, at the level of location. For attributes scored at the respondent level, the mean score (N = 16) is given.

When all attributes were included in the measure of susceptibility, vulnerability to fishing was greater at Ahus than Karkar (*Z* = −2.2; *p* = 0.031) (Figure 8). However, when only the four catchability attributes were included in the measure of susceptibility, vulnerability to aggregation fishing did not differ significantly between locations (*Z* = −1.6; *p* = 0.156) (Figure 8). This occurs because differences in the four catchability attributes tend to cancel each other out, such that fisher knowledge and selectivity are higher at Ahus but the reverse is true for availability and encounterability (Figure 9). With the exception of preference for the six species, the socioeconomic attributes used in the PSA scored more highly for Ahus than Karkar (Figure 9). The score for fishing effort was higher at Ahus since fishers at that location averaged 93 hours of fishing each month compared to 58 hours at Karkar. Dependency on fishing was also higher, with more than 80% of fishers reporting fishing as their primary livelihood compared to 12.5% at Karkar. The majority of fishers at Ahus also reported that they use scare lines for catching snappers, ensuring that habitat impacts were also comparatively high at that location. Moreover, fishers from Ahus regularly accessed markets of neighbouring villages (neighbouring islands and the northern coast of Manus) and the provincial capital, whereas fishers from Karkar generally traded locally or occasionally in neighbouring villages on Karkar. Ownership rights and customary reef closure measures still existed at Karkar, but at Ahus fishers reported during interviews that the customary closure was no longer being respected or complied with. Consequently, including attributes relating to socioeconomic drivers resulted in a greater susceptibility of aggregations to fishing at Ahus compared to Karkar.

## Discussion

Supportive of previous research findings in Papua New Guinea [6], we found that fisher knowledge of aggregating behaviour varied between our two case study communities. While this finding is unsurprising in a country of such high cultural and socioeconomic diversity, our study makes a contribution by also highlighting the influence of local context in structuring knowledge within communities. Thus, the relatively high heterogeneity in knowledge at Ahus related to rights of access among fishers and genders to the main habitats of the relatively large reef system. By contrast, knowledge was more homogenous at Karkar and the sole source of variation was primary gear type. Though knowledge of aggregation location and timing are prerequisites for exploitation, factors that drive fishing effort will ultimately determine their susceptibility to fishing since knowledge, by itself, does not ensure that fishers will seek to maximize their extraction from the fishery [46]–[47]. Overfishing may be related to distance to markets [2], while the overexploitation of spawning aggregations has been attributed to the emergence of commercial markets for aggregating species [29]. Social norms operating outside customary tenure may also constrain fishing pressure [47]–[48]. However, our study objectives required a trade-off between the qualitative interviews that are required to explore social norms and quantitative approaches involving larger sample sizes that were needed for statistical inference of the factors relating to knowledge. Additional research exploring how knowledge is structured within communities will be important for assessing the role of social norms or customary management practices, such as *tambu* areas, in regulating fishing effort.

### Factors associated with heterogeneity in fisher knowledge of aggregating behaviour

Fisher knowledge of aggregating behaviour was particularly heterogeneous at Ahus. Since variation at Ahus related to gender and access to reef habitats, it appears to stem from the decentralised tenure system that specifies ownership rights to space, species, gear and the techniques for using gears among kinship groups [22], [48]–[49]. An understanding of how knowledge is structured and maintained among kinship groups is important since the breakdown of customs and the spread of knowledge can lead to increased fishing pressure on aggregations [6]. By contrast, marine tenure arrangements at Karkar allow for relatively higher mobility of fishers between gears and fishing grounds [22]. With less specialisation in specific gears or habitats, knowledge was less structured at Karkar compared to Ahus, as indicated by the relatively low amount of variation explained by the redundancy analysis. Heterogeneity in knowledge was unrelated to dependency on fishing, which at Ahus resulted from the fact that fishing was the primary livelihood for 80% of fishers, encompassing both genders and fishers with differing right of access. Though our objective was to explore local sources of variation in knowledge, dependency on fishing may explain why fishing effort was higher and fishers used more efficient gears (i.e. scare lines) at Ahus.

Variation in fisher knowledge of aggregating behaviour differed among the three families of reef fish. Compared to emperors and groupers, knowledge of aggregations of the two snappers was greater and relatively homogenous. Snapper aggregation locations were perceived by fishers as spatially predictable and were associated with particular features of the reef, corresponding with empirical evidence for these species [25], [50]. Of our six study species, snappers generally had the highest reported catch rates, presumably since they are schooling species and are relatively abundant, often forming an important component of reef fisheries catch in many parts of Papua New Guinea and other regions [51]–[52].

Heterogeneity in fisher knowledge relating to grouper spawning aggregations was structured by access rights or gear type, depending on location. Grouper spawning aggregations mainly form on outer reef slopes and channels [9], [11], [53]. Since around one third of fisher respondents at Ahus, including all females and some male fishers, were limited to using nets or fishing in the large lagoon, they are therefore unlikely to have developed knowledge of this behaviour. Gear use also plays a role in knowledge acquisition [38] and fishers using spearguns at Karkar held greater knowledge of grouper spawning aggregations, possibly benefitting from the direct observation of fish behaviour that this gear affords. However, few grouper spawning aggregations may exist at Karkar since the scales of migration (approximately 10–25 km; [15], [54]) that these species are known to undertake in attending spawning aggregations are larger than the linear extent (<5 km) of the reef fished [36].

As with groupers, heterogeneity in fisher knowledge of emperor behaviour also stemmed from variable rights of access among fishers. Thus, lagoon fishers at Ahus developed specialist knowledge of the behaviour of *L. harak*, a generally solitary species common to that habitat [6]–[7], which forms spawning aggregations and small groups for non-reproductive functions [55]–[56]. Reports of *L. lentjan* spawning in aggregations were also limited to Ahus fishers, and are consistent with anecdotal reports of this behaviour from other countries [57]. Contrasting with its congener, *L. lentjan* primarily feed in deeper water [58] and heterogeneity in knowledge emerged on Ahus as the species was mainly known to male fishers who can access the outer reefs. Fishers from both locations generally perceived emperors as being relatively mobile and of lower spatial predictability than groupers or snappers, which concurs with scientific evidence [59].

Our study was a first step in quantitatively exploring the factors that influence local ecological knowledge of reef fish aggregating behaviour within communities, but was limited to a small number of socioeconomic indicators that reflect the contemporary context of the two communities. Consequently, we did not quantify important historical aspects of these communities and the role of oral histories in transferring knowledge, which may have influenced the patterns observed in our data. For example, though we pooled respondents from the two study villages of Karkar Island in our analyses, knowledge of grouper spawning aggregations was higher (by 52%, based on the sum fisher knowledge index for both grouper species) among Muluk fishers than neighbouring fishers from Wadau. Since fishers exhibit similar dependency on fishing, this finding may reflect the differing historical context of two communities, which may have settled on the coast at different times [22].

Comparing several locations in Melanesia, Hamilton et al. (2004) [6] found that the Titan communities from southern Manus held the richest bodies of knowledge pertaining to grouper spawning aggregation sites, which had accumulated over generations. The knowledge base that supports the complex tenure systems of Ahus and neighbouring Ponam Island also extends over many generations and is likely reinforced through cultural mechanisms such as initiation rights for certain fishing practices [37], [48]. Thus, knowledge of aggregating behaviour at Ahus has likely been retained through such mechanisms and is presumably limited to clans or kinship groups that can access those resources. While it would have been informative to stratify sampling of respondents by clans or kinship groups, this poses difficulties owing to the often complex relationships in communities such as Ahus [22]. Adding further factors in the analysis would also have required more interviews to be conducted than our resources permitted, since RDA is a constrained ordination analysis that is sensitive to the number of factors relative to sample size. Though our RDA results should be interpreted with caution, since the number of factor levels slightly exceeded the recommended number based on sample size, unconstrained analyses (i.e. principal components analysis) yielded similar relationships between fisher knowledge and socioeconomic indicators.

### Susceptibility of reef fish populations to aggregation fishing

This study demonstrated the utility of PSA in examining how attributes relating to catchability and socioeconomic drivers of fishing effort influence the susceptibility of reef fish populations to aggregation fishing. Given that experts with access to scientific information have scored susceptibility indictors in previous applications of PSA [30], [31], our study is also novel in that information to score indicators was sourced directly from resource users. Such an approach is more applicable to the many coral reef fisheries that lack fisheries and ecological information.

Knowledge of fish aggregating behaviour is commonly utilized by fishers to improve catchability and returns from a fishery [4]. It is therefore appropriate to incorporate fisher knowledge as an indicator of susceptibility to fishing in risk analyses involving aggregating species, especially as it varies among communities [6]. At Karkar, encounterability was higher and the main reef habitats were available for access by all fishers, including the outer reef slopes that are the typical aggregation habitat of at least three of our study species (*E. fuscoguttatus*, *L. gibbus* and *P. areolatus* [53]). However, knowledge of aggregating behaviour was less well developed than on Ahus. The selectivity of gears for many species was greater on Ahus, particularly in their use of a form of muro-ami to target snappers, whereby scare lines and nets are used to corral fish into an enclosed space where they are taken by speargun. This fishing technique is highly efficient for shoaling and schooling fish such as *L. gibbus* [43], as evidenced by the higher catch rates at Ahus. Thus, after combining fisher knowledge with availability, encounterability and selectivity, study populations were assessed as equally susceptible to aggregation fishing at Ahus and Karkar using the catchability approach of Hobday et al. (2011) [30].

An advantage of PSA is that the susceptibility attributes included can be adapted to local contexts or issues of importance. For example, Patrick et al. (2010) [31] developed 22 susceptibility index indicators in a PSA, including attributes relating to socioeconomic drivers of fishing pressure (termed ‘management’ attributes). This proved important to our analysis since the use of catchability attributes alone did not separate study locations, whereas the inclusion of socioeconomic drivers identified a greater potential for overfishing of aggregating populations at Ahus. These drivers are known indicators of fishing pressure and resource depletion [2], [34]. Moreover, conservation benefits provided by the *tambu* at Ahus will have been lost with the recent breakdown in this governance system [22]. It is therefore beneficial to understand the socioeconomic factors that drive demand for marine resources and may lead to greater targeting of reef fish aggregations.

PSA and other semi-quantitative approaches are, however, sensitive to the assumptions made in developing indicators [30]. A number of assumptions had to be made in our application for aggregation-based fisheries. For example, our proxy for encounterability assumed that if a species appeared in a fisher's catch, then the gears used by that fisher were equally likely to encounter the species in aggregations if conditions of availability were met. This may be justified if aggregations did not form at depths beyond those typically fished by the gear, which would presumably affect fishers using gears that are generally constrained to shallower water (i.e. spearguns and nets) than fishers using lines. Our measure of availability was coarse but necessary in a data-poor context with limited time for observations on the spatial distribution of fishing effort. The use of CPUE to estimate selectivity was also subject to uncertainty, given the numerous factors that affect this parameter, and essentially constituted a measure of gear efficiency for a species rather than selectivity [33]. Further development of indicator-based frameworks for aggregation-based reef fisheries may improve on our methods for estimating susceptibility to fishing.

An additional caveat in the use of indicator-based approaches is that they can be overly reductionist in attempting to simplify complex socio-ecological systems. Consequently, PSA can be combined with more detailed social, economic or ecological research to better understand the management implications of more complex interactions that indicators fail to capture. It is ideally applied as a participatory risk assessment tool for supporting communication, promoting understanding, building consensus and prioritizing actions as part of community-based management planning. In our application of this tool, five populations were identified to be at medium risk from aggregation fishing. However, a participatory application of PSA in the two communities may have yielded different results. For example, resource users could develop their own indicators or choose to weight indicators according to their own priorities [31]. Though PSA has a strong basis in theoretical and empirical evidence [33], [60], its validity as a predictive tool requires robust assessment [31]. We were unable to validate our application of this tool since biomass estimates for the study populations are absent. However, multispecies reef fish biomass was lower at Ahus than Karkar, both at the time of the interviews (D. Feary, pers. comm.) and in 2002 [37], [61], which is likely indicative of the higher fishing effort at Ahus [22] and may also reflect the status of our study populations.

To conclude, heterogeneity in fisher knowledge relating to reef fish aggregating behaviour will be influenced by social, economic and cultural factors that are specific to the local context. Understanding how knowledge is structured within a community will be important if customary practices for conservation, such as *tambu* areas, are to be supported by working with relevant kinship groups. While knowledge alone does not imply that fishers will maximize extraction from a fishery, shifts in socioeconomic drivers may serve to increase fishing pressure. For example, a breakdown in ownership rights and resulting spread of knowledge among kinship groups has been identified as a cause for concern in relation to pressure on spawning aggregations in PNG [6]. At Ahus, the relatively high susceptibility of aggregations to fishing, caused by a combination of high dependency on fishing, access to larger markets and loss of the *tambu* areas, would be exacerbated if the system of ownership rights also weakened. However, aggregations forming for purposes other than reproduction are also predictable and may be highly susceptible to fishing if efficient gears are used [8]. The findings of this study therefore call for a holistic approach to assessing the risks posed by fishing on reef fish aggregations, one that is grounded in the principals of fisheries science and emerging social-ecological thinking [34].
